# Extraction Optimization and Bioactivity of Polysaccharides from *Ganoderma leucocontextum* Spores

**DOI:** 10.3390/ph18020241

**Published:** 2025-02-11

**Authors:** Siying Peng, Yanghui Ou, Yali Zhang, Hongliang Yao, Wen-Hua Chen

**Affiliations:** 1 School of Pharmacy and Food Engineering, Wuyi University, Jiangmen 529020, China; 13528399622@163.com; 2 Guangdong Key Laboratory of Animal Conservation and Resource Utilization, Institute of Zoology, Guangdong Academy of Sciences, Guangzhou 510260, China; ouyh0807@gmail.com (Y.O.); zhangyl@giz.gd.cn (Y.Z.)

**Keywords:** three-phase partitioning, polysaccharide, *Ganoderma leucocontextum* spore, anti-inflammation, anti-aging

## Abstract

**Background:** Oxidative stress is associated with the occurrence and progress of aging. Natural polysaccharides have attracted considerable attention in the field of antioxidants and anti-aging products due to their superior biological activity and low toxicity. Ga*noderma leucocontextum* is primarily found in the Tibetan plateau region and is classified as a subspecies of *Ganoderma*. Known as the famous white *Ganoderma*, it is a precious food and medicine that has potent biological activity, including antitumor, hypoglycemic, and immune regulation. Since available resources are limited, there are few studies on the spore of *Ganoderma leucocontextum*. **Methods:** In this work, a polysaccharide (named GLSP) was extracted from the spore of *Ganoderma leucocontextum* using a fast, simple, efficient, and environmentally friendly extraction process: the three-phase partitioning (TPP) method. **Results:** The extraction condition was optimized under the Box–Behnken design (BBD): ratio of the solute to the solvent, 1:21.126 (*w*/*v*); (NH_4_)_2_SO_4_ concentration, 30% (*w*/*v*); ratio of the slurry to tert-butanol, 1:1.945 (*v*/*v*); and shaking temperature, 54.136 °C. Furthermore, a polysaccharide termed GLSP-A1 was purified from GLSP by column chromatography. The basic physicochemical properties were analyzed by molecular weight, Fourier transform infrared spectroscopy, monosaccharide composition, and scanning electron microscopy. **Conclusions:** GLSP-A1 down-regulated the expression of the pro-inflammation cytokines interleukin-6 and interleukin-1β, indicating favorable in vitro anti-inflammatory properties. In vivo, the effect of GLSP-A1 on aging was examined using the Caenorhabditis model. The results showed that GLSP-A1 reduced reactive oxygen species levels and lipofuscin accumulation. In general, these findings improve our understanding of the chemical content and bioactivity of a polysaccharide from *Ganoderma leucocontextum* spore and highlight the possibility of GLSP-A1 being utilized in dietary supplements for its anti-aging properties.

## 1. Introduction

Aging is an inevitable natural process that can lead to a variety of age-related diseases [1]. The accumulation of lipofuscin, the denaturation of functional proteins, and damage to cell membrane structure have been attributed to the effects of reactive oxygen species (ROS) and are known to be associated with aging [2]. *Caenorhabditis elegans* is a model organism that is widely used in anti-aging research because it has genetic and physiological characteristics similar to those of humans, such as a short life cycle, making it convenient for experimental operation.

*Ganoderma leucocontextum* is classified under *Basidiomycota*, *Agariomycetes*, *Polyporales*, *Ganodermataceae*, and the *Ganoderma* genus. It is primarily found in the Tibetan plateau region and is also known as white *Ganoderma* or Tibetan *Ganoderma lucidum*. Formerly considered a subspecies of *Ganoderma lucidum*, it was classified as a new species of *Ganoderma* in 2015 [3]. *Ganoderma leucocontextum* is rich in bioactive compounds, including polysaccharides, triterpenoids, and proteins [4]. *Ganoderma leucocontextum* has wide application prospects such as hypoglycemic, antitumor, antihyperlipidemic, and immunomodulation properties (Table 1). Due to limited resources, there has been little research on the extraction and efficacy of spores from *Ganoderma leucocontextum*.

Polysaccharides have several beneficial effects, such as anti-inflammatory [17], antioxidant [18], antitumor [19], and immunomodulatory activity [20]. Natural polysaccharides have drawn more interest because of their possible biological function and minimal toxicity. Fungi used in edible medicine is a significant natural source of bioactive polysaccharides [21].

Common methods of polysaccharide extraction include hot water extraction, microwave-assisted extraction, supercritical fluid extraction, and enzyme-assisted extraction derived from hot water extraction. Although crude polysaccharides can be simply extracted by these traditional methods, the extracted polysaccharides are often mixed with proteins and pigments, requiring further purification and resulting in low efficiency and utilization rate of raw materials. The chemical reagents and process auxiliaries used in traditional extraction methods not only incur high costs but also damage polysaccharides, leading to environmental pollution and other harm.

TPP is a safe, efficient, widely applicable, and low-cost extraction method. Compounds such as oils, proteins, enzymes, polysaccharides, and lipid-soluble pigments can be extracted from raw materials by TPP. It can also meet the separation of a variety of components at the same time to increase the value of raw materials [22]. It is composed of an aqueous solution of (NH_4_)_2_SO_4_ mixed with an organic solvent, primarily *tert*-butanol. After mixing and centrifugation, the whole component is divided into three different layers: an upper layer of lipid-soluble compounds (*tert*-butanol), a lower layer of water-soluble compounds (polysaccharides and (NH_4_)_2_SO_4_), and a protein-rich layer in the middle [23]. In previous studies, TPP technology was rarely used to extract and isolate polysaccharides from *Ganoderma lucidum* spore, and further purification and determination of its physicochemical properties and anti-inflammatory activity were also rare.

Therefore, in this study, TPP technology was used to directly isolate a polysaccharide (GLSP) from *Ganoderma lucidum* spores, and then, the response surface method (RSM) was used to optimize the TPP extraction process. A neutral polysaccharide (GLSP-A1) was purified by column chromatography. The physicochemical properties and anti-aging activity of GLSP-A1 were determined.

## 2. Results and Discussion

### 2.1. Traditional and TPP Extraction of Polysaccharides from Ganoderma lucidum Spores

In the traditional extraction method, the spores of Ganoderma leucocontextum were mixed with purified water according to a certain ratio of material to liquid and then decocted at 80 °C for 4 h. The supernatant was concentrated and lyophilized to obtain the crude polysaccharide from white Ganoderma lucidum spores, named GLAP-HW. The polysaccharide obtained by the three-phase extraction method was named GLSP-TPP.

#### 2.1.1. Extraction Rates, Uronic Acid, and Protein Content of GLSP-HW and GLSP-TPP

The extraction rates, uronic acid, and protein content of GLSP-HW and GLSP-TPP are shown in Table 2.

#### 2.1.2. Anti-Inflammatory Activities of GLSP-HW and GLSP-TPP

**Figure 1:**
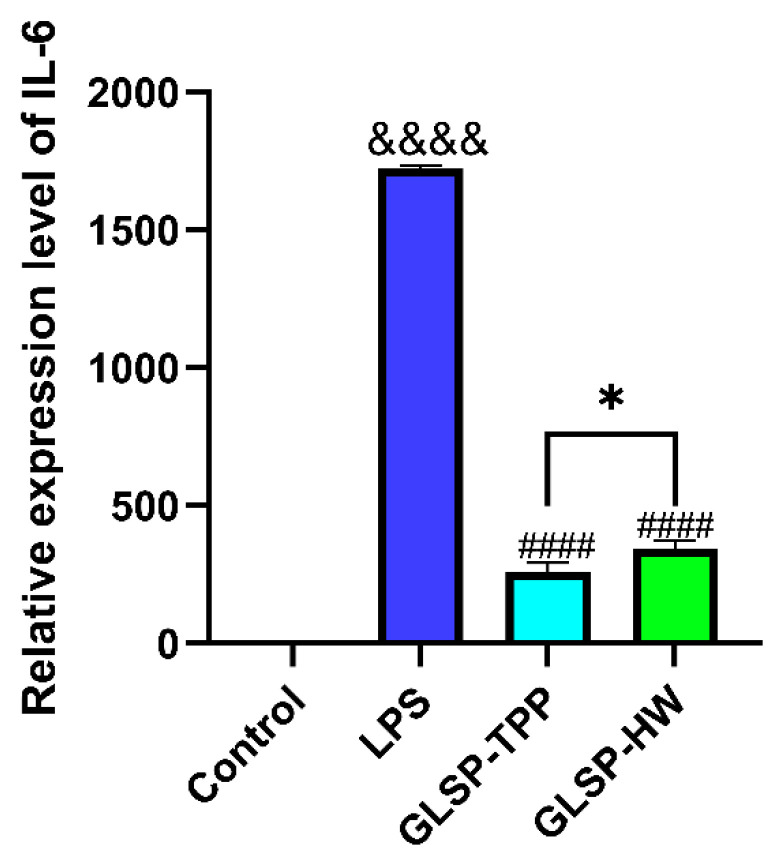
Effect of GLSP-HW and GLSP-TPP on LPS-induced IL-6 relative expression in RAW264.7 macrophages compared to the blank group (^&&&&^ *p* < 0.001), the LPS group (^####^ *p* < 0.001), and the GLSP-HW group (* *p* < 0.05). The above values are expressed as mean ± SD (n = 3).

The anti-inflammatory activities of GLSP-HW and GLSP-TPP are shown in Figure 1.

#### 2.1.3. Infrared Spectroscopy of GLSP-HW and GLSP-TPP

**Figure 2:**
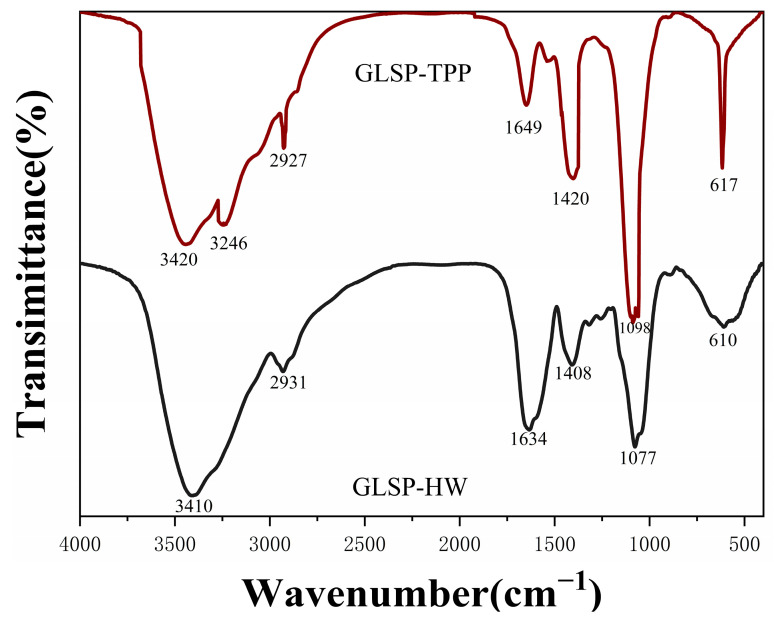
Infrared spectroscopy of GLSP-TPP and GLSP-HW.

Infrared spectroscopy plays a key role in the rational analysis of polysaccharides and is mainly used to identify and analyze specific groups in molecules. The infrared spectroscopy results of GLSP-HW and GLSP-TPP are shown in Figure 2.

The peaks at 1634 cm^−1^ represent the carbonyl C=O stretching vibrations of terminal sugar residues or uronic acid [24]. This result indicates the presence of more uronic acids in the GLSP-HW, which was consistent with the results listed in Table 1. The peak of GLSP-TPP at 1418 cm^−^^1^ is the result of the variable angle vibration of CH [25]. GLSP-TPP has prominent and sharp peaks at 1098 cm^−^^1^, indicating that GLSP-TPP has more adipose ether bonds [26].

In summary, we know that GLSP-HW and GLSP-TPP are different due to different extraction processes. GLSP-TPP had higher extraction yield, lower uronic acid content and protein content, and better anti-inflammatory activity compared to GLSP-HW. Therefore, the three-phase extraction of white Ganoderma lucidum spore polysaccharides is more advantageous. Next, we will further study the three-phase extraction of Ganoderma lucidum spore polysaccharides. For convenience, we will simply write GLSP-TPP as GLSP.

### 2.2. Extraction Process Optimization

#### 2.2.1. Single-Factor Experiment on GLSP

**Figure 3:**
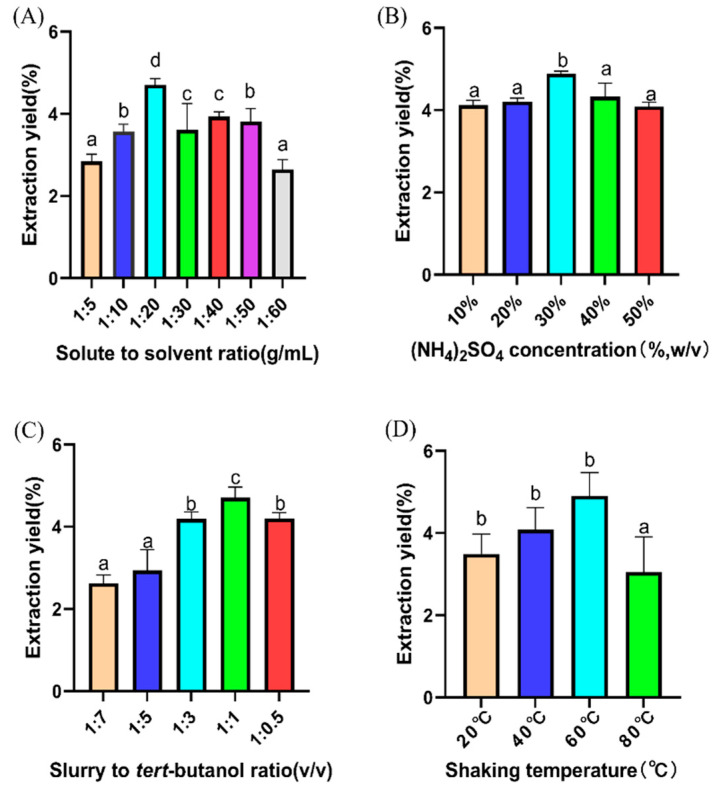
Single-factor experiment on the extraction yield of GLSP. (**A**) Solute-to-solvent ratio. (**B**) (NH_4_)_2_SO_4_ concentration. (**C**) Slurry-to-*tert*-butanol ratio. (**D**) Shaking temperature. Different letters (a–d) indicate that the difference was statistically significant (*p* < 0.05).

The effects of the solute-to-solvent ratio, (NH_4_)_2_SO_4_ concentration, ratio of the slurry to *tert*-butanol, and shaking temperature on the extraction rate of GLSP were studied (Figure 3). When the ratio of the solute to the solvent was 1:20, the concentration of sodium sulfate was 30%, the ratio of the slurry to tert-butanol was 1:1, and the shaking temperature was 60 °C, the extraction yield of GLSP reached the peak.

As shown in Figure 3A, the extraction of GLSP first increased and then decreased when the solute-to-solvent ratio ranged from 1:5 to 1:60 (g/mL). This may be due to the fact that the mass transfer driving force of polysaccharides reaches its maximum at 1:20 (g/mL) [27].

Figure 3B shows the effect of (NH_4_)_2_SO_4_ concentration on the extraction yield of GLSP. NH_4_^+^ and SO_4_^2−^ stabilize the intermolecular interactions of biological macromolecules and are closely related to the “salting out” [28] effect. At 10–30% (*w*/*v*) (NH_4_)_2_SO_4_, the salting out effect was the positive driving force for the increase in GLSP yield. At 40–50% (*w*/*v*) (NH_4_)_2_SO_4_, excess (NH_4_)_2_SO_4_ aggravated the “salt-out” effect, and GLSP production gradually decreased.

*Tert*-butanol concentrates fat-soluble compounds in the upper phase. As shown in Figure 3C, when the ratio of the slurry to tert-butanol was reduced from 1:7 to 1:0.5, an effective synergistic effect was generated between tert-butanol and (NH_4_)_2_SO_4_, which increased the extraction rate of GLSP, and the extraction rate of GLSP reached the highest level at 1:1.

The extraction yield of GLSP gradually increased from 40 °C to 60 °C with shaking and decreased after 60 °C (Figure 3D). This is due to the increase in the temperature of the TPP system, where a large number of hydroxyl groups in polysaccharide molecules are exposed and more hydrogen bonds are formed, thus increasing the hydrophilicity of polysaccharides and resulting in an increase in the concentration of the bottom-phase GLSP.

#### 2.2.2. Response Surface Methodology

According to the single-factor experiment, the solute-to-solvent ratio, slurry-to-*tert*-butanol ratio, and shaking temperature were among the main variables that significantly impacted the extraction yield of GLSP-A1. Consequently, in order to design and optimize process variables and obtain the greatest yield (Y) of GLSP-A1, this work employed the three-factor and three-level BBD of the RSM. The experimental data and technology variables are displayed in Table 3. When the solute-to-solvent ratio was 1:20 (g/mL), the slurry-to-*tert*-butanol ratio was 1:1 (*v*/*v*), and when the shaking temperature was 60 °C, the Y reached the maximum of 5.14%. The experimental results were subjected to multiple regression analysis, which produced the following second-order polynomial equation.

Yield = 5.35 + 0.3108 ×A + 0.1850 ×B − 0.3743 × C + 0.1735 × AB − 0.2325 × AC − 0.2934 × BC − 1.81 × A^2^ − 0.9293 *× B^2^ − 0.7612 × C^2^

An ANOVA was used for the significance and precision type of the mode. A summary of the statistics is presented in Table 4. The more significant the model is, the smaller the probability *p*-value. The coefficient of determination (R^2^) of the fitted model was 0.9908, indicating that only 0.92% of the total variation was not explained by the model. The adjusted coefficient of determination (Adj-R^2^) was 0.9789, suggesting that the significance of this model was high. The monomial coefficients (A, B, and C), quadratic term coefficients (A^2^, B^2^, and C^2^), and interaction term coefficients (AC and BC) were significant, whereas the coefficients of the other parameters were not significant. There was no significant lack of fit (*p* > 0.05), which proves the validity of the model; the low coefficient of variation (C.V. = 4.81%) demonstrates that the model possesses strong dependability.

Therefore, the interaction of the solute-to-solvent ratio (A) and slurry-to-*tert*-butanol ratio (B) with shaking temperature (C) has a significant effect on the extraction of GLSP and should be fully considered.

**Figure 4:**
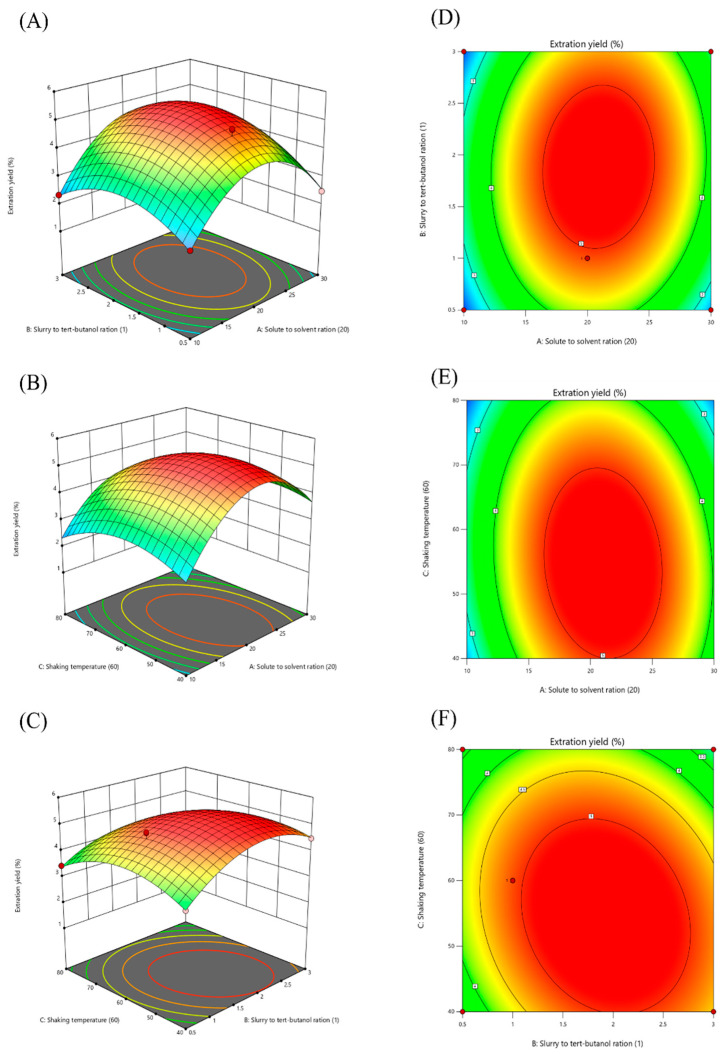
Response surface (**A**–**C**) and contour plots (**D**–**F**) showing the effect of the solute-to-solvent ratio (A, g/mL), slurry-to-*tert*-butanol ratio (B, mL/mL), and shaking temperature (C, °C) on the extraction yield of GLSP (Y, %).

The response surface and higher line plots were plotted using the Design-Expert 13 software. Fixing one of the three variables as the median value, the effect surface plots of the extraction rate were plotted. The generated 3D response surface and 2D contour plots are shown in Figure 4A–F and reveal the relationship and interaction between these process variables. From Figure 4A–C, it can be seen that the C effect surface was steeper compared to the B direction, indicating that the shaking temperature had a greater effect on the extraction rate than the solute-to-solvent ratio and slurry-to-*tert*-butanol ratio. The interaction between these factors was significant based on the shapes of the contour lines and the steepness of the response curves shown in Figure 4A,D–F. An increase in two variables, A1 and A2, resulted in an initial increase in the extraction yield of GLSP-A1, followed by an observed decrease. The maximum Y of GLSP was achieved when the ratio of the solute to the solvent was 1:20, the ratio of the slurry to tert-butanol was 1:1, and the shaking temperature was 60 °C. The denser the elliptic contour plot, the more significant the interaction between the variables. Thus, the interaction between A and C is stronger than the other interaction variables.

According to the analysis of variance (Table 4) and response surface diagram (Figure 4A–F), the numerical optimization results show that the Y conditions of GLSP under this system are as follows: solute-to-solvent ratio of 1:21.126 (g/mL), slurry-to-tert-butanol ratio of 1:1.945, and oscillation temperature of 54.136 °C. Using the above conditions, the Y of GLSP predicted by the model was 5.432%. Considering the actual experimental conditions, the experimental conditions close to the predicted standard were used for verification; that is, the solute-to-solvent ratio was 1:20, the slurry-to-tert-butanol ratio was 1:1.9, and the shaking temperature was 55 °C. The final extraction rate of GLSP obtained was 5.3 ± 0.027%. Experiment Y is close to the predicted value, which verifies the validity of the BBD model.

### 2.3. Purification of GLSP

**Figure 5:**
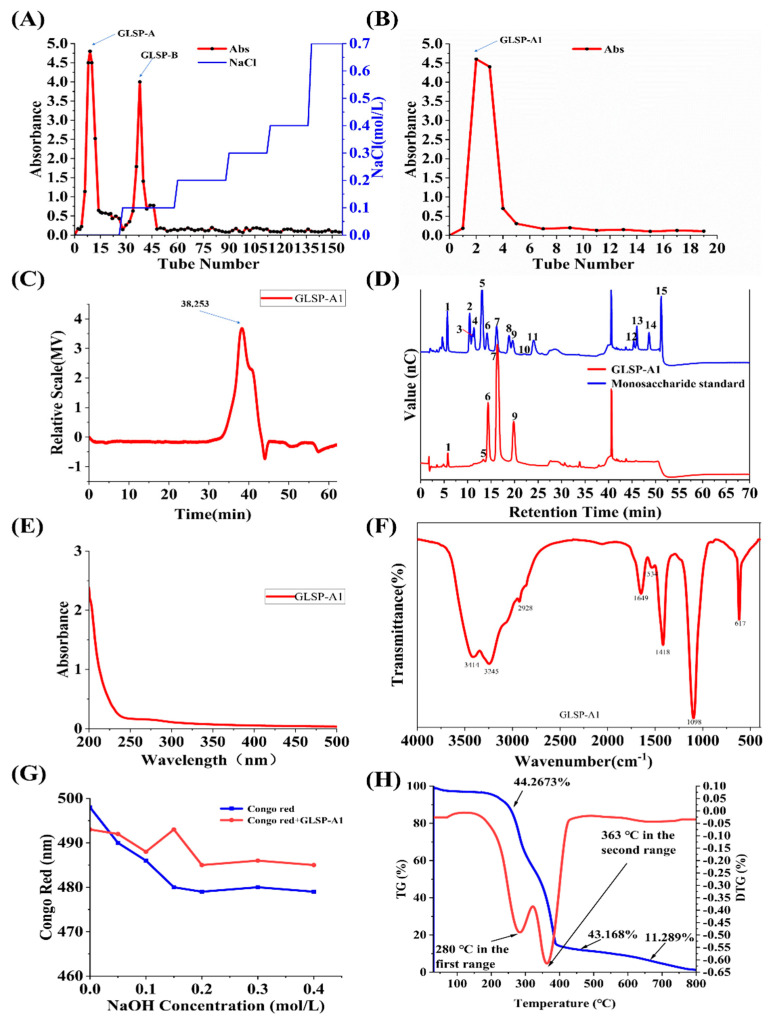
(**A**) DEAE-52 anion exchange column chromatography elution curve. (**B**) Experdex 75 gel osmotic column elution curve. (**C**) HPGPC spectrum and peak Mw of GLSP-A1. (**D**) HPLC chromatograms of standard monosaccharides and GLSP-A1: 1, Fuc; 2, GalN; 3, Rha; 4, Ara; 5, GlcN; 6, Gal; 7, Glc; 8, Xyl; 9, Man; 10, Fru; 11, Rib; 12, GalA; 13, GulA; 14, GlcA; and 15, ManA. (**E**) UV spectra of GLSP-A1. (**F**) FT-IR of GLSP-A1. (**G**) Congo Red experimental analysis of GLSP-A1. (**H**) Thermal analysis of the GLSP-A1.3.3.8. FT-IR analysis.

GLSP was eluted with ultrapure water and 0.1M NaCl using DEAE-52 anion column exchange chromatography. Then, GLSP-A and GLSP-B were obtained after dialysis and lyophilization (Figure 5A). Their yield was 30% and 17.8%, and their total sugar content was 84.5% and 43.54%, respectively. GLSP-A was further purified in a gel permeation column Experdex 75 (2.6 × 60 cm) to obtain GLSP-A1 (Figure 5B).

### 2.4. Characterization of GLSP-A1

#### 2.4.1. Total Sugars, Uronic Acid, and Protein Content

Total sugars, uronic acid, and protein content of GLSP and GLSP-A1 are shown in Table 5.

#### 2.4.2. Molecular Weight Determination

HPGPC was used to determine the molecular weight (Mw), number average molecular weight (Mn), and polydispersity index (Mw/Mn) of GLSP-A1. The major Mw of GLSP-A1 was 15,750 Da (Figure 5C). The molecular weight of GLSP-A1 was much lower than that of polysaccharides from Ganoderma lucidum spores (GLSP, main peaks: 81,679 Da) previously reported by Wen et al. [29]. Low-molecular-weight polysaccharides have the ability to penetrate biofilms without inducing immune system stress, which may result in increased biological activity [30]. GLSP-A1 may have good biological activity.

#### 2.4.3. Monosaccharide Composition Analysis of GLSP-A1

HPIC was used to ascertain the monosaccharide composition of GLSP-A1 (Figure 5D). The results showed that it consisted of fucose, glucosamine hydrochloride, galactose, glucose, and mannose with a mole ratio of 0.03:0.002:0.201:0.497:0.27. Our study used polysaccharides from mushroom Lingzhi spores (GLSP-I, glucose composition) that were different from those reported by Wen et al. [31].

#### 2.4.4. Ultraviolet–Visible Spectrum Analysis

The absence of absorption peaks in the UV–VIS spectrum at 260 nm and 280 nm confirms the absence of proteins or nucleic acids in GLSP-A1 (Figure 5E) [32].

#### 2.4.5. FT-IR Analysis

As seen in (Figure 5F), the IR spectra of GLSP-A1 spans from 4000 cm^−1^ to 500 cm^−1^. There is a wide peak near 3414 cm^−1^, which produces strong and widespread -OH strength vibrations due to intermolecular and intramolecular hydrogen bonds [33]. The feeble peak observed at about 2928 nm-1 was related to the -CH stretching vibration, which included -CH, -CH2, and -CH3 [34]. Two powerful and pointed peaks detected at 1649 cm^−1^ and 1418 cm^−1^ are usually generated by C=O vibrations, confirming the presence of carbonyl groups [35]. The characteristic absorption of ether bonds is 1098 cm^−1^. It is both the normal IR signal of glucans and the characteristic absorption peak of sugars [26].

#### 2.4.6. Congo Red Assay of GLSP-A1

The maximum absorption wavelength changes of Congo Red and GLSP-A1 in NaOH solutions at varying concentrations (0 to 0.4 M) are displayed in Figure 5G. In contrast to Congo Red, GLSP-A1’s maximum absorption wavelength did not significantly shift with an increase in NaOH concentration, suggesting that the compound lacks a triple helix structure.

#### 2.4.7. Thermal Analysis of GLSP-A1

The thermal stability of polysaccharides is usually measured by thermogravimetry (TG) and derivative thermogravimetry (DTG) methods. TG and DTG test curves show the three weight loss stages of GLSP-A1 (Figure 5H). In the initial weight loss stage of GLSP-A1 (30–250 °C), the weight loss rate is about 44.2673%, indicating the water evaporation of GLSP-A1 [36]. From 250 °C to 450 °C during the second stage of weight loss, the weight loss rate is about 43.168%, which may be the result of the depolymerization of the polysaccharide structure [37]. The third weight loss stage was from 450 °C to 800 °C, and the weight loss rate was about 11.289%, which may be caused by the decomposition of macromolecular or inorganic impurities [38]. The DTG curve showed that GLSP-A1 had a large weight loss rate (0.475%) at 280 °C in the first stage and a maximum weight loss rate (0.6%) at 363 °C in the second stage, among which 363 °C was the most significant loss point in the whole weight loss process.

#### 2.4.8. Scanning Electron Microscopy (SEM) Assay

**Figure 6:**
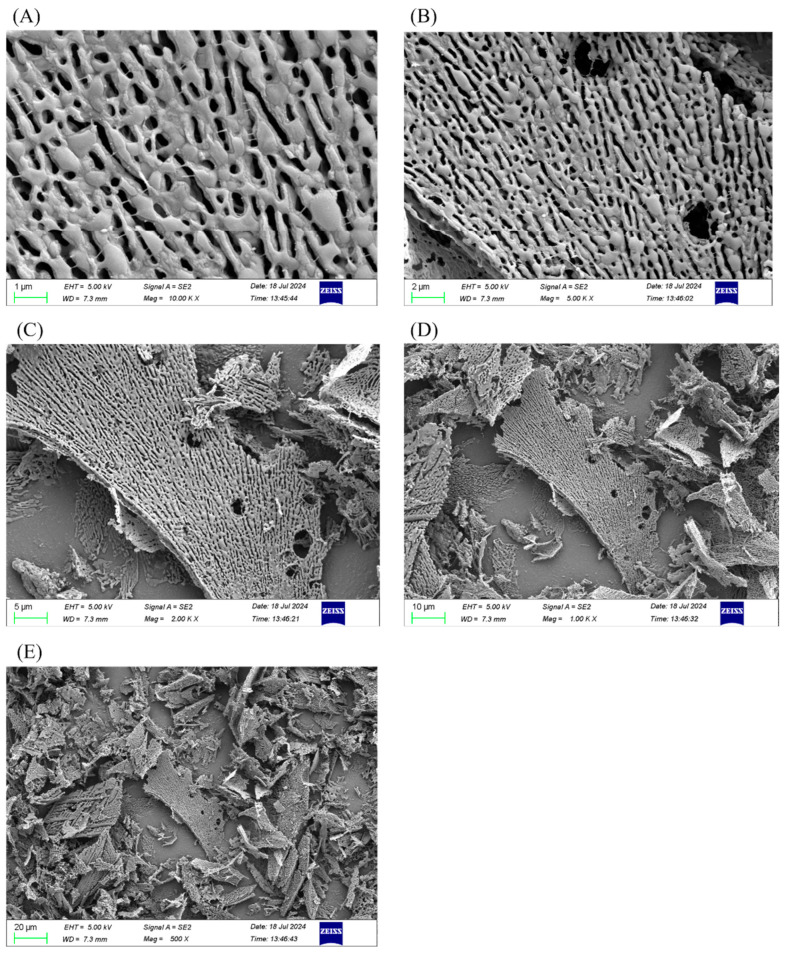
Scanning electron micrographs of GLSP-A1 at magnification ((**A**): 10,000×, (**B**): 5000×, (**C**): 2000×, (**D**): 1000×, and (**E**): 500×). The lyophilized samples were coated with a thin layer of gold and photographed by a scanning electron microscope.

A scanning electron microscope was used to examine the surface morphology of GLSP-A1 (Figure 6A–E). SEM technology can intuitively show the spatial distribution, arrangement, and interaction with other molecules of polysaccharides and other microscopic information, which provides an important basis for the structural study of polysaccharides.

The apparent morphology of the polysaccharide GLSP-A1 gradually changed from lamellar to porous, forming a loose network structure. These forms may be formed by the association and assembly of some polysaccharide molecules through intra-chain and inter-chain hydrogen bonds or other secondary bonds. Presenting a reticular structure, this may be due to the greater distance between the chains caused by the higher negative charge density, which promotes the expansibility and hydrophilicity of the structure [39].

### 2.5. In Vitro Anti-Inflammatory Activity of GLSP-A1

**Figure 7:**
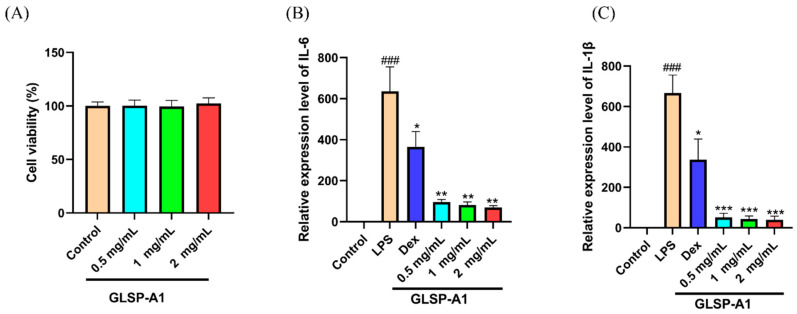
(**A**) Effect of GLSP-A1 on the viability of RAW264.7 macrophages. (**B**) Effect of GLSP-A1 on LPS-induced IL-6 relative expression in RAW264.7 macrophages. (**C**) Effect of GLSP-A1 on LPS-induced IL-β relative expression in RAW264.7 macrophages compared to the blank group (### *p* < 0.001) and the LPS group (* *p* < 0.05, ** *p* < 0.01, and *** *p* < 0.001). The above values are expressed as mean ± SD (n = 3).

Small-molecule peptides or glycoproteins, named cytokines, are synthesized and secreted by a variety of tissue cells, mainly immune cells. In addition to their ability to mediate cell–cell interactions, cytokines also perform a multitude of biological functions, including controlling immunological response, wound healing, proliferation and differentiation of cells, cell development, and tumor growth and expansion [40]. Immune cells cause local and systemic inflammatory responses that help clear pathogens; growth factors such as the vascular endothelial growth factor and epidermal growth factor stimulate the proliferation of fibroblasts, endothelial cells, etc., promoting angiogenesis and wound healing. In response to LPS stimulation, macrophages release a range of inflammatory mediators and proteins, including tumor necrosis factor-α (TNF-α), interleukin-1β (IL-1β), interleukin-6 (IL-6), and interleukin-10 (IL-10) [41]. As illustrated in Figure 7, we assessed the anti-inflammatory efficacy of GLSP-A1 in RAW264.7 macrophages through the quantification of IL-6 and IL-β expression. DEX and GLSP-A1 markedly reduced the expression of IL-6 and IL-β in comparison to the LPS group. Cell viability was unaffected by GLSP-A1 at doses ranging from 0.5 to 2 g/mL (*p* > 0.05) (Figure 7A). The anti-inflammatory effect was enhanced as the concentration of GLSP-A1 increased from 0.5 to 2 g/mL (Figure 7B,C). GLSP-A1 decreased the expression of IL-6 and IL-1 β in a dose-dependent manner. GLSP-A1 inhibits the synthesis and release of a variety of inflammatory mediators. By increasing the activity of antioxidant enzymes, it reduces oxidative stress, thereby inhibiting the production of inflammatory factors. The polysaccharide from *Ganoderma lucidum* spores (GLSP) reported by Wen et al. [29] also had the ability to down-regulate the expression of IL-6, IL-1β, and NO.

### 2.6. In Vitro Anti-Aging Experiment in C. elegans

#### 2.6.1. GLSP-A1 Reduce ROS Accumulation in *C. elegans*

**Figure 8:**
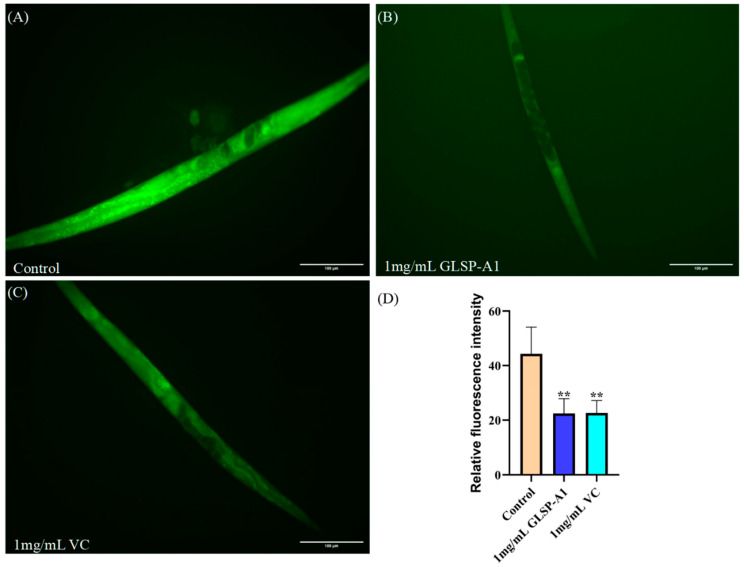
(**A**–**C**) ROS fluorescence levels in the nematodes of each group. (**D**) Relative fluorescence intensity of ROS in nematodes compared to the blank group (** *p* < 0.05). The above values are expressed as mean ± SD (n = 3).

ROS is closely related to the aging of organisms. On the one hand, excessive reactive oxygen species can lead to cell damage and senescence, and on the other hand, appropriate reactive oxygen species can act as signaling molecules and participate in a variety of physiological processes within cells, including cell growth, differentiation, and apoptosis [42]. DCFH-DA (2′,7′-dichlorofluorescein diacetate) is a commonly used fluorescent probe for the detection of intracellular reactive oxygen species (ROS) levels. As shown in Figure 8, the level of fluorescence accumulation of ROS was reduced in nematodes treated with 1 mg/mL GLSP-A1 and 1 mg/mL VC compared to the control. It is worth noting that the ROS level of GLSP-A1 at a concentration of 1 mg/mL was significantly lower than that of the control by 49.34%(** *p* < 0.05). The evidence is clear that polysaccharides from *Ganoderma leucocontextum* spores reduce ROS accumulation in nematodes, thereby delaying the extent of nematode senescence.

#### 2.6.2. GLSP-A1 Reduce Lipofuscin Accumulation in *C. elegans*

**Figure 9:**
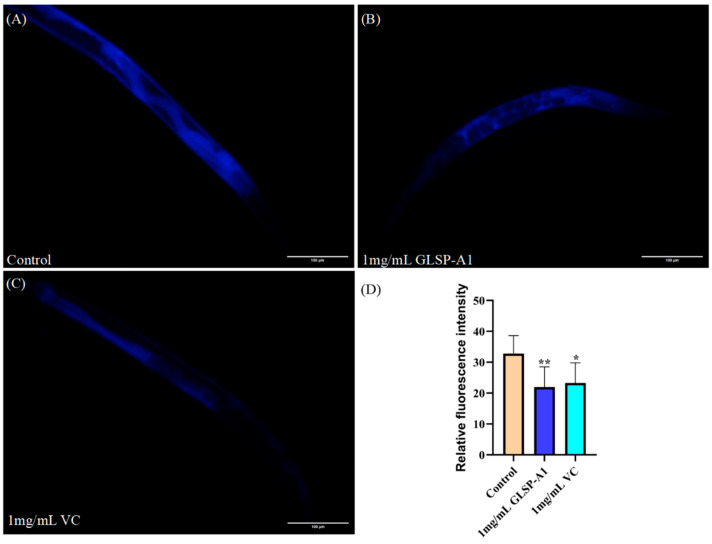
(**A**–**C**) Blue fluorescence produced by lipofuscin in the nematodes of each group under fluorescent irradiation. (**D**) Effects of 1 mg/mL GLSP-A1 and 1 mg/mL VC on lipofuscin in nematodes compared to the blank group (* *p* < 0.1, ** *p* < 0.05). The above values are expressed as mean ± SD (n = 3).

Lipofuscin is a residue of organelle fragments and lipid peroxidation products that have not been fully digested in autophagolysosomes. Lipofuscin accumulation increases with age, and it is commonly found in nerve, heart muscle, liver, and other tissue cells; it also occurs during the formation of senile plaques on the surface of the skin [43]. Its accumulation will lead to the decline of cell function and affect the energy metabolism of cells, resulting in an insufficient energy supply of cells, and may eventually lead to cell death and tissue degradation, which is one of the important manifestations that are closely related to the aging process [44]. Moreover, lipofuscin contains a large number of oxidized lipids; these oxidized lipids can produce free radicals, further promote the oxidative stress of cells, and accelerate the aging process of cells [45]. Therefore, reducing the accumulation of lipofuscin may help delay aging [46]. Under oxidative stress with H_2_O_2_, cells will produce a large amount of oxygen free radicals, which will attack the unsaturated fatty acids in the cells and trigger lipid peroxidation to form lipofuscin. Under fluorescence microscopy, lipofuscin in nematodes can be observed to display spontaneous blue fluorescence. Compared to the control, the fluorescence accumulation level of lipofuscin was decreased in nematodes treated with 1 mg/mL GLSP-A1 and 1 mg/mL VC (Figure 9). VC and GLSP-A1 have antioxidant effects that can help neutralize free radicals and reduce oxidative stress, thus potentially reducing lipofuscin production. The 1 mg/mL GLSP-A1 lipofuscin level was significantly decreased by 33.15% compared to the blank group (** *p* < 0.05).

The purified *Ganoderma leucocontextum* spore polysaccharide GLSP-A1 has the characteristics of high total sugar content, low molecular weight, and monosaccharide-like composition and structure, which are closely related to its biological activity. There is a close relationship between anti-inflammatory and anti-aging. Chronic inflammation is a long-term low-grade inflammatory state that accelerates cellular damage and the aging process [47]. After cell senescence, pro-inflammatory cytokines will be secreted to promote the occurrence of chronic inflammation and form a vicious cycle [48]. Anti-inflammation has become one of the important strategies for anti-aging. By reducing chronic inflammation, it can slow down cell damage and organ aging processes and reduce the risk of age-related diseases [49]. In the whole study, we explored the mechanism of the aging effect of *Ganoderma leucocontextum* spore polysaccharides through an in vitro anti-inflammatory experiment and an in vivo anti-aging nematode experiment. GLSP-A1 significantly decreased the expression of IL-6 and IL-β and showed good anti-inflammatory activity. GLSP-A1 can down-regulate the levels of reactive oxygen species and lipofuscin in *C. elegans*, indicating that GLSP-A1 has good anti-aging activity. In conclusion, we believe that the anti-aging mechanism of *Ganoderma leucocontextum* spore polysaccharides may be related to the interaction of its anti-inflammatory activity with the ability to down-regulate ROS and lipofuscin, thus playing an anti-aging role.

## 3. Materials and Methods

### 3.1. Materials and Chemicals

Ganoderma leucocontextum spores were obtained from Tibet Milin Red Sun Tibetan Medicinal Material Science and Technology Development Co., Ltd., Milin County, Tibet, China. The cell culture medium (RAW264.7 mouse macrophage cell) was obtained from Wuhan Pricella Biotechnology Co, Ltd. (Wuhan, China), and qPCR reagents (#Q711-02) were obtained from Nanjing Vazyme Biotechnology Co., Ltd. (Nanjing, China). Lipopolysaccharide (LPS (#L2880)) and dexamethasone (Dex (#D1756)) were obtained from Merck Co., Ltd. (Darmstadt, Germany). All the other chemicals and solvents were of laboratory grade and were used directly.

### 3.2. Traditional Extraction and TPP Extraction of Polysaccharides from Ganoderma lucidum Spores

#### 3.2.1. Traditional Extraction of Ganoderma Leucocontextum Spore Polysaccharide (GLSP-HW)

Traditional hot water extraction is a widely used method for polysaccharide extraction. The principle is that most polysaccharides have a greater solubility in hot water. The usual practice is to perform decoction in hot water for 2–6 h. The traditional extract of *Ganoderma leucocontextum* spore polysaccharides was named GLSP-HW.

#### 3.2.2. TPP of Ganoderma Leucocontextum Spore Polysaccharide (GLSP-TPP)

To obtain a solute-to-solvent ratio of 1:20 (g/mL), 0.5 g *Ganoderma leucocontextum* spore was put into a 50 mL round-bottom flask, and 10 mL ultrapure water was added to make a solution, followed by decoction for four hours at 80 °C in a water bath and centrifugation at 4000 rmp for 10 min. Then, the supernatant liquid was transferred to a 50 mL centrifuge tube, after which 3 g (NH_4_)_2_SO_4_ (20%, *w*/*v*) and 10 mL of *tert*-butanol (volume ratio of the slurry to *tert*-butanol was 1:1) were added and the mixture was shaken for 10 min at 40 °C in a tank with constant temperature oscillation. After shaking and centrifuging at 5000 rmp for 10 min, the mixed solution formed three clear phases. The upper organic phase was collected by needle and moved to a fresh 50 mL centrifuge tube and kept cold (4 °C). The lower-layer solution ((NH_4_)_2_SO_4_ salt solution and water-soluble polysaccharides) was collected with a needle, and the middle layer (free protein) was stored at 4 °C. The lower solution was dialyzed with distilled water (dialysis bag MWCO: 3.5 kDa) for 48 h to remove salts and small molecular substances. Then, the solution was frozen and dried to obtain the polysaccharide GLSP-TPP, named GLSP for short.

#### 3.2.3. Extraction Rates, Uronic Acid, and Protein Content of GLSP-HW and GLSP-TPP

The extraction rate, which is calculated as the percentage of the weight of the polysaccharide extracted from the raw material divided by the weight of the raw material, is a crucial measurement to evaluate the impact of polysaccharide extraction. The extraction yield (Y) was computed using the following formula.

$$Y(\%)=\frac{M_{1}}{M_{2}}$$

where M_1_ is the weight of the lyophilized extract (g) and M_2_ is the weight of Ganoderma leucocontextum spores (g).

Using the sulfate–carbazole method [50] and the Bradford method [51], the uronic acid and protein contents of the GLSP-HW and GLSP-TPP were determined, respectively; galacturonic acid and bovine serum albumin (BSA) were used as standards, respectively.

#### 3.2.4. Anti-Inflammatory Activities of GLSP-HW and GLSP-TPP

The groups included an administration group (1 mg/mL GLSP-HW and GLSP-TPP + 1 µg/mL lipopolysaccharide (LPS)), an LPS stimulation group (1 µg/mL), and a normal control group (without LPS stimulation). Following a 4 h induction with 1 µg/mL LPS, RNA was collected and cDNA was produced by real-time fluorescence quantitative PCR, following Vazyme’s method [52].

#### 3.2.5. Infrared Spectroscopy of GLSP-HW and GLSP-TPP

The specimen was prepared using the KBr disc procedure, and the FT-IR spectrum analysis was recorded using a 6700 Nicolet Fourier transform infrared spectrophotometer (Thermo Co., Madison, WI, USA) within the 4000 to 400 cm^−1^ range. GLSP-HW and GLSP-TPP were analyzed using infrared spectra.

### 3.3. Extraction Process Optimization

#### TPP Single-Factor Experiment

According to the extraction steps of GLSP 3.2.1, the effects of the solute-to-solvent ratio (g/mL), the concentration of (NH_4_)_2_SO_4_ (%, *w*/*v*), the ratio of slurry to *tert*-butanol (mL, *v*/*v*), and shaking temperature (°C) on the extraction rate of GLSP were investigated. The setting levels of each factor are shown in Table 6.

Using the Box–Behnken design, we examined the response surface experiment based on the findings of the single-factor experiment, with three factors and three levels, namely the solute-to-solvent ratio (A_1_, g/mL), the ratio of the slurry to *tert*-butanol (A_2_, *v*/*v*), and shaking temperature (A_3_, °C) (Table 7); 17 experiments were conducted with 5 central points. The three factors were prescribed as three grades, coding low, medium, and high values of −1, 0, and +1, respectively. The extraction rate (%) of the GLSP is the response output value.

### 3.4. Purification of Polysaccharide from Ganoderma Leucocontextum Spores

GLSP (1.5 g) was reconfigured in 50mL ultrapure water and eluted on a diethylaminoethyl cellulose 52 (DEAE-52) anion column (5.5 × 50 cm) with twice the column volume of ultrapure water and 0.1–0.7 M NaCl solution at a flow rate of 2 mL/min. Using the phenol–sulfuric acid technique [53], the total carbohydrate content of the elution fraction (8 mL/tube) was determined. Tubes with the same elution peak were collected. Then, the fraction obtained from ultrapure water was further purified on an Experdex 75 gel permeation column (2.6 × 60 cm). The elution peak was obtained when the column was eluted with ultrapure water at a flow rate of 1.0 mL/min. The purified Ganoderma Leucocontextum spore polysaccharide (GLSP-A1) was obtained by dialysis and lyophilization.

### 3.5. Characterization of GLSP-A1

#### 3.5.1. Extraction Yield, Total Sugars, Uronic Acid, and Protein Content

Using the phenol–sulfuric acid method, sulfate–carbazole method, and Bradford method [51], the GLSP-A1’s total sugar, uronic acid, and protein contents were determined, respectively.

#### 3.5.2. Molecular Weight Determination

To guarantee homogeneity, the polysaccharide GLSP-A1 (5 mg) was dissolved in 1 mL of purified water and then passed through a 0.22 μm filtration membrane. Mixing GLSP-A1 with purified water ensured that the sample was sufficiently dissolved to form a homogeneous solution. This helps to improve the accuracy and repeatability of the HPGPC test. The 0.22 μm filtration membrane can effectively filter out macromolecular impurities, proteins, microorganisms, etc., in the solution, ensuring the purity of the sample entering the HPGPC system and avoiding the contamination or interference of these impurities on the chromatographic column and detector. High-performance gel permeation chromatography (HPGPC), using an Agilent LC-10A high-performance liquid chromatography (HPLC) platform with a gel column (BRT105-104-102; 8 × 300 mm) and a refractive index detector (RID), was used to measure the molecular weight. The differential refractive detector (RID) determines the sample molecular weight by measuring the change in the refractive index between the sample flow path and the reference flow path when GLSP-A1 samples are analyzed in a high-performance gel permeation chromatography (HPGPC) system. After entering the HPGPC system, the GLSP-A1 samples were separated in the chromatographic column according to the molecular size. The separated sample fractions were sequentially entered into the detection cell of the RID. When the sample solution passes through the detection cell, it causes a deflection of the beam because its refractive index is different from that of the pure solvent, and this deflection is detected by the photodiode and converted into an electrical signal. The strength of the electrical signal is proportional to the concentration of the sample, thus obtaining the concentration information of the sample. Combined with the retention time of the chromatogram, the calibration curve can be established by the traditional correction method to calculate the relative molecular weight of GLSP-A1. The chromatographic conditions were as follows: 0.01 M NaCl; flow rate of 0.5 mL/min; column temperature of 40 °C; and injection volume of 20 μL. Agilent GPC software was used to evaluate the data. As a result, the molecular weight of GLSP-A1 was determined using the calibration curve created based on the retention time of molecular standards and the peak shape of the HPGPC chromatogram [54].

#### 3.5.3. Monosaccharide Composition Analysis

High-performance ion exchange chromatography (HPIC) was used to determine the monosaccharide analyses. A pulse ampere detector (PAD) and a Dionex CarbopacTM PA20 column (3 × 150 mm, Thermo Fisher Scientific, Waltham, MA, USA) were used in the procedure. Reference sugars such as rhamnose, d-ribose, arabinose, xylose, inositol, allose, mannose, glucose, and galactose were used to study the components of monosaccharides. With the area normalization approach, the relative molar ratios of monosaccharides were determined based on the chromatogram [55].

#### 3.5.4. Ultraviolet–Visible Spectrum Analysis

Using an ultraviolet–visible (UV–VIS) spectrophotometer (NP80 Touch, Implen, Munich, Germany), the GLSP-A1 was scanned in the 200–500 nm range at a concentration of 0.1 mg/mL.

#### 3.5.5. Fourier Transform Infrared (FT-IR) Spectroscopy Analysis

Fourier transform infrared (FT-IR) spectroscopy was used to analyze GLSP-A1.

#### 3.5.6. Congo Red Assay of GLSP-A1

A solution of GLSP-A1 with a concentration of 1 mg/mL and 80 µM Congo Red was mixed sequentially with NaOH solutions of varying concentrations (0, 0.05, 0.1, 0.15, 0.2, 0.3, and 0.4 mol/mL). After a 5 min standing period, the UV–VIS (NP80 Touch, Implement, Munich, Germany) spectrophotometer was used to detect the solution’s maximum absorption wavelength within the 400–600 nm range.

#### 3.5.7. Thermal Properties

A Netzsch STA 449F3 apparatus (Selb, Germany) was used for the thermal gravimetric (TG), differential thermal gravity (DTG), and differential scanning calorimetry (DSC) investigations of GLSP-A1. A nitrogen atmosphere was applied to an Al_2_O_3_ crucible containing 8 mg of GLSP-A1. The temperature was increased from 25 °C to 800 °C at a rate of 10 °C/min [56].

#### 3.5.8. Scanning Electron Microscopy (SEM) Assay

Using a scanning electron microscope (Sigma-300, Carl Zeiss AG, Oberkochen, Germany), the surface morphology of the GLSP-A1 was observed [57].

### 3.6. In Vitro Anti-Inflammatory Activity of GLSP-A1

#### 3.6.1. Cell Culture

The American Type Culture Collection (Manassas, VA, USA) provided the murine macrophage RAW 264.7 cell line, which was cultivated in Dulbecco’s Modified Eagle’s Medium with 10% fetal bovine serum and 1% penicillin–streptomycin. The cells were cultured at 37 °C with 5% CO_2_ in a humidified environment.

#### 3.6.2. Cell Viability Assay

The Cell Counting Kit (CCK) reduction colorimetric assay was used to assess the vitality of the cells. RAW264.7 cells were seeded in 96-well plates. Next, varying quantities of GLSP-A1 were applied (0, 0.5, 1, and 2 µg/mL). Following a 24 h incubation period, 10 μL of CCK was applied to each well and kept for four hours. After removing the supernatants, the formazan crystals were dissolved using 100 μL of dimethyl sulfoxide. Using a microplate reader (Molecular Devices Corporation, San Jose, CA, USA), the absorbance was measured at 450 nm. The experiment was conducted three times, and cell survival was reported as a percentage.

#### 3.6.3. Cytokine Measurement

The groups included an administration group (0.5, 1, and 2 mg/mL GLSP-A1 + 1 µg/mL lipopolysaccharide (LPS)), an LPS stimulation group (1 µg/mL), and a normal control group (without LPS stimulation). A dexamethasone group (DEX, 1 µM) was used as a positive control. Following a 4 h induction with 1 µg/mL LPS, RNA was collected and cDNA was produced by real-time fluorescence quantitative PCR, following Vazyme’s method [52].

### 3.7. Evaluating the Impact of GLSP-A1 on Aging in C. elegans

#### 3.7.1. Cultivation of *C. elegans*

All *C. elegans* were grown on nematode growth medium (NGM) plates and inoculated with *E. coli* OP50 at 20 °C. The *E. coli* was cultured for 12 h at 37 °C. Eggs were obtained using a bleaching solution, and M9 buffer was used to wash the eggs. After 48 h of synchronization, L4 nematodes were prepared for the subsequent assays.

#### 3.7.2. Determining the Levels of Reactive Oxygen Species (ROS)

Synchronized L4 nematodes were divided into a blank group, a 1 g/mL GLSP-A1 administration group, and a 1g/mL Vc positive control group. Following incubation 48 h after administration, the nematodes were subjected to 10 mmol/mL H_2_O_2_ treatment for 15 min. All the worms were gathered and washed three times using M9 buffer. To analyze the ROS content, worms were exposed to a solution of 10 mM DCFH-DA fluorescent probe dye (Biyuntian, Shanghai, China) for 30 min at a temperature of 37 °C. The fluorescence microscope (EVOS, Thermometer Fisher Scientific, MA, USA) and ImageJ software (NIH, Bethesda, MD, USA) were employed to determine and examine the relative intensity of ROS fluorescence.

#### 3.7.3. Lipofuscin Accumulation Assay

Synchronized L4 nematodes were selected onto an NGM plate containing 1 mg/mL GLSP-A1 and OP50, and the control group was treated with 1 mg/mL Vc as the solvent control. After 2 days of administration, the nematodes were treated with 10 mmol/mL H_2_O_2_ for 15 min and then transferred to a centrifuge tube containing M9 buffer. A tube containing nematodes was centrifuged at 3000 rmps for 2 min to precipitate nematodes. After the supernatant was abandoned, the nematodes were placed on a 2% agarose plate. They were examined by fluorescence microscopy. We used bright-field and dark-field microscopy to observe its shape, and then take pictures. We used ImageJ to measure fluorescence. The light intensity was analyzed statistically using GraphPad Prism 8.

## 4. Conclusions

Compared to traditional extraction methods, TPP provides a straightforward, effective, and environmentally friendly approach to extract polysaccharides from *Ganoderma leucocontextum* spores. We optimized the extraction process of GLSP and obtained the purified neutral polysaccharide GLSP-A1, which was composed of fucose, glucosamine hydrochloride, galactose, glucose, and mannose. The bioactivity evaluation showed that GLSP-A1 had favorable anti-inflammatory and antioxidant efficacy. The results can promote the application of this precious ingredient in anti-aging diseases, advancing the use of *Ganoderma leucocontextum* spores in pharmaceuticals and nutritional supplements.

## Figures and Tables

**Table 1:** Active ingredients extraction from *Ganoderma leucocontextum.*

Extraction Solvent	Constituents	Efficacy	Reference
Ethanol	Terpenoids	Hypoglycemic	[5]
Ethanol	Lanostane triterpene	Hypoglycemic	[6]
Ethanol	Triterpenoids	Hypoglycemic	[7]
Hot water	Heteropolysaccharide	Hypoglycemic	[8]
Ethanol	Merolterpenoids	Hypoglycemic	[9]
Ethanol	Lanostane triterpene GL22	Anticancer	[10]
Ethanol	Ganoderiol F	Anticancer	[11]
Ethanol	Meroterpenoids ganomycin I	Antihyperlipidemic	[12]
Hot water	Water-soluble polysaccharide GLP-3	Immunomodulation	[13]
Hot water	Water-soluble polysaccharide GLP-1	Immunomodulation	[14]
Hot water	Water-soluble polysaccharide GLP-1	antioxidant	[15]
Ethanol	Terpenoids	Neuroprotective effect	[16]

**Table 2:** Extraction rates, uronic acid, and protein content of GLSP-HW and GLSP-TPP.

	Extraction Rate (%)	Uronic Acid Content (%)	Protein Content (%)
GLSP-HW	1.1	2.828	2.9697
GLSP-TPP	3.06	0.9167	2.5247

**Table 3:** Experimental data and technology variables of the RSM.

Entry	Coded Variable Levels			Yield (%)
	*A* ^a^	*B* ^a^	*C* ^a^	
1	1:10	1:1	40	2.07
2	1:20	1:3	80	3.16
3	1:30	1:1	80	2.12
4	1:20	1:1	60	4.68
5	1:20	1:0.5	80	3.44
6	1:30	1:1	40	3.02
7	1:10	1:1	80	2.1
8	1:1	1:3	60	3.28
9	1:20	1:0.5	40	3.54
10	1:20	1:1	60	5.1
11	1:20	1:3	40	4.48
12	1:30	1:0.5	60	2.48
13	1:10	1:3	60	2.34
14	1:20	1:1	60	4.74
15	1:20	1:1	60	5.14
16	1:20	1:1	60	4.84
17	1:10	1:0.5	60	2.32

^a^*A*, *B*, and *C* denote the solute-to-solvent ratio (g/mL), slurry-to-*tert*-butanol ratio (mL/mL), and shaking temperature (°C), respectively.

**Table 4:** ANOVA of the RSM.

Source	GLSP					
	Sum of Squares	df	Mean Square	F-Value	*p*-Value	Significant
Model	20.80	9	2.31	83.40	<0.0001	***
A: Solute-to-solvent radio	0.6705	1	0.6705	24.19	0.0017	**
B: Slurry-to-*tert*-butanol ratio	0.2738	1	0.2738	9.88	0.0163	*
C: Shaking temperature	0.9724	1	0.9724	35.08	0.0006	**
AB	0.1421	1	0.1421	5.13	0.0580	
AC	0.2162	1	0.2162	7.80	0.0268	*
BC	0.4064	1	0.4064	14.66	0.0065	*
A^2^	13.81	1	13.81	498.38	<0.0001	***
B^2^	1 25	1	1.25	45.18	0.0003	**
C^2^	2.44	1	2.44	88.04	<0.0001	***
Residual	0.1940	7	0.0277			
Lack of Fit	0.0188	3	0.0063	0.1432	0.9289	not significant
Pure Error	0.1752	4	0.0438			
Cor Total	21	16				
R^2^ = 0.9908, Adj-R^2^ = 0.9789, R^2^ pre = 0.9719, and C.V.% = 4.81						

* Visually indicates the significance of the coefficients (*p*-value): *** *p* < 0.001, ** *p* < 0.01, and * *p* < 0.05.

**Table 5:** Total sugars, uronic acid, and protein content of GLSP and GLSP-A1.

	Total Sugar Content	Uronic Acid Content	Protein Content
GLSP	35.37%	0.92%	0.79%
GLSP-A1	90.5%	Nd	Nd

Nd: Not detected.

**Table 6:** Single-factor test.

Solute-to-Solvent Ratio (g/mL)	(NH_4_)_2_SO_4_ Concentration (%, *w*/*v*)	Slurry-to-*tert*-Butanol Ratio (*v*/*v*)	Shaking Temperature (°C)
1:5	10%	1:7	20
1:10	20%	1:5	40
1:20	30%	1:3	60
1:30	40%	1:1	80
1:40	50%	1:0.5	
1:50			
1:60			

**Table 7:** BBD test design and factor level.

Level	Factors		
	A_1_ Solute-to-Solvent Ratio (g/mL)	A_2_ Slurry-to-*tert*-butanol Ratio (*v*/*v*)	A_3_ Shaking Temperature (°C)
−1	1:10	1:3	40
0	1:20	1:1	60
1	1:30	1:0.5	80

## Data Availability

Data are contained within the article.

## Abbreviations

BBD	Box–Behnken design
CCK	Cell Counting Kit
DEAE-52	diethylaminoethyl cellulose 52
DEX	dexamethasone
DTG	derivative thermogravimetry
FT-IR	Fourier transform infrared
GLSP-HW	a polysaccharide extracted from Ganoderma leucocontextum by traditional extraction
GLSP-TPP	a polysaccharide extracted from Ganoderma leucocontextum by three-phase partitioning
GLSP-A1	a polysaccharide purified from GLSP by column chromatography
HPGPC	high-performance gel permeation chromatography
HPIC	high-performance ion exchange chromatography
HPLC	high-performance liquid chromatography
IL-6	interleukin-6
IL-10	interleukin-10
IL-1β	interleukin-1β
LPS	lipopolysaccharide
Mn	molecular number
Mw	molecular weight
Mw/Mn	polydispersity index
PAD	pulse ampere detector
SEM	scanning electron microscopy
TG	thermogravimetry
TPP	three-phase partitioning
TNF-α	tumor necrosis factor-α
UV–VIS	ultraviolet–visible
